# A Significant Reduction in the Frequency of HIV-1 Drug Resistance in Québec from 2001 to 2011 Is Associated with a Decrease in the Monitored Viral Load

**DOI:** 10.1371/journal.pone.0109420

**Published:** 2014-10-08

**Authors:** Hugues Charest, Florence Doualla-Bell, Régis Cantin, Donald G. Murphy, Linda Lemieux, Bluma Brenner, Isabelle Hardy, Daniela Moisi, Ernest Lo, Jean-Guy Baril, Mark A. Wainberg, Michel Roger, Cécile Tremblay

**Affiliations:** 1 Laboratoire de santé publique du Québec/Institut national de santé publique du Québec, Sainte-Anne-de-Bellevue, Québec, Canada; 2 Département de microbiologie, infectiologie et immunologie, Faculté de médecine, Université de Montréal, Montréal, Québec, Canada; 3 Department of Medicine, Division of Experimental Medicine, McGill University, Montréal, Québec, Canada; 4 McGill AIDS Center, Lady Davis Institute, Jewish General Hospital, McGill University, Montréal, Québec, Canada; 5 Centre hospitalier de l’Université de Montréal (CHUM), Montréal, Québec, Canada; 6 Department of Epidemiology, Biostatistics and Occupational Health, McGill University, Montréal, Québec, Canada; 7 Clinique du Quartier Latin, Montréal, Québec, Canada; University of Pittsburgh, United States of America

## Abstract

**Background:**

HIV drug resistance represents a major threat for effective treatment. We assessed the trends in the frequency of drug resistance mutations and the monitored viral load (VL) in treatment-naïve (TN) and treatment-experienced (TE) individuals infected with HIV-1 in Québec, Canada, between 2001 and 2011.

**Methods and Findings:**

Resistance data were obtained from 4,105 and 5,086 genotypic tests performed on TN and TE patients, respectively. Concomitantly, 274,161 VL tests were carried out in the Province. Changes over time in drug resistance frequency and in different categories of VL were assessed using univariate logistic regression. Multiple logistic regression was used to evaluate associations between the rates of certain mutations and antiretroviral prescriptions. From 2001 to 2011, the proportion of undetectable VL test results continually increased, from 42.1% to 75.9%, while a significant decrease in the frequency of resistance mutations associated with protease inhibitors [PI (from 54% to 16%)], nucleoside [NRTI (from 78% to 37%) and non-nucleoside reverse transcriptase inhibitors [NNRTI (from 44% to 31%)] was observed in TE patients. In TN individuals, the overall frequency of transmitted drug resistance was 13.1%. A multiple logistic regression analysis indicated that the introduction of co-formulated emtricitabine/tenofovir or emtricitabine/tenofovir/efavirenz was positively associated with the decrease of the frequency of the M184I/V mutations observed overtime (p = 0.0004).

**Conclusions:**

We observed a significant decrease in the frequency of drug resistance mutations in TE patients, concomitant with a decrease in the proportion of patients with detectable viremia. These findings may be related to both the increased potencies and adherence to therapy associated with newer antiretroviral regimens. Nevertheless, our data demonstrate that broad use of antiretrovirals does not increase the level of circulating drug resistant variants.

## Introduction

In view of the recent findings of the efficacy of antiretroviral therapy (ART), not only for the health benefit of individuals [1], [2] but also to prevent HIV transmission [2], programs to expand the use of ART have been implemented in several countries. Several challenges remain in regard to stopping the spread of HIV through the use of ART in infected individuals. One is the emergence of antiretroviral drug resistance, which has been widely documented. However, with more potent antiretroviral regimens, treatment failure rates have been steadily declining in recent years.

Trends in monitored viral load and frequency of HIV-drug resistance are good indicators of the effectiveness of clinical and public health interventions throughout the cascade of care, from diagnosis to viral suppression [3]–[6]. Our study aimed to analyze the frequency and putative determinants of HIV-1 drug resistance in patients in the province of Québec from 2001 to 2011, as monitored through the use of two surveillance program databases.

## Materials and Methods

### Provincial programs for the follow-up of HIV-1 infected individuals

In Québec, ART, HIV-1 VL assays and genotyping for resistance testing are universally available. The province wide program for HIV-1 viral load testing was introduced in 1997. The test is usually prescribed on a quarterly basis for individuals with an HIV-1 diagnosis. Assays are centralized in three tertiary-care hospital laboratories. In October 2001, a provincial program for drug resistance testing was initiated via a network of three laboratories. Clinical indications for requesting HIV genotyping include therapeutic failure (treatment-experienced group), perinatal transmission, pregnant women who test positive for HIV, and primary HIV infection. The latter is defined as a newly diagnosed HIV infection where a documented seroconversion occurred within the six months prior to collection of the diagnostic specimen. Since 2004, HIV genotyping has also been offered to chronically infected individuals in order to detect transmitted antiretroviral resistance mutations prior to ART-initiation (baseline test). Both newly diagnosed and chronically infected individuals were part of the treatment-naïve (TN) group in the study. A clinical justification has not been a prerequisite for testing samples with a VL>400 copies/ml.

Tests are requested by treating clinicians as part of the clinical follow up of HIV-1 infected individuals. Clinical samples are identified with nominal information in order to ensure proper tracking in medical records at the hospital. Results are de-identified at hospital laboratories before being submitted to the LSPQ databases for analyses. Results and anonymized socio-demographic data for HIV-1 VL and drug resistance testing programs have distinct, non-nominal databases that are centralized at the provincial reference laboratory (Laboratoire de santé publique du Québec; LSPQ). Treating physicians and laboratory staff have no access to LSPQ databases, from which the data presented here are derived.

Before 2003, 77.6% of HIV genotyping requests were justified based on therapeutic failure as a clinical indication (data not shown). This proportion gradually decreased over the years to 30.1% in 2011, when baseline monitoring for patients initiating ART was the main reason for testing (44.7%). The proportion of tests performed for pregnant women (<5%) and primary infections (<15%) remained stable overtime and only a few cases (n = 4) of vertical transmission of HIV were reported during this 11 year period. Tests performed for other than indicated clinical justifications or for undisclosed reasons represented 9.5% to 14% of tests, respectively, and were therefore not included in analyses comparing TN and TE individuals.

### Methods for viral load measurements

Monitored viral load was used as a metering to monitor province-wide HIV burden and treatment outcomes, (http://www.cdc.gov/hiv/topics/surveillance/resources/factsheets/viral_load.htm). From 2001 to June 2010, HIV-1 VL was determined using Bayer’s VERSANT bDNA 3.0 assays (Diagnostics Division, Bayer Corp., Tarrytown, N.Y.), with a limit of detection of 50 RNA copies/ml of plasma. From June to December 2010, VL assays were carried out using the Abbott Real-Time HIV-1 kit, with a limit of detection of 40 RNA copies/ml.

### Methods for HIV-1 drug resistance testing

The compilation of genotypic resistance results, based on the first test performed per patient and per year, generated a total of 9,191 sequences over the period of the study. A total of 4,347 sequences presented with mutations associated with any ARV drug resistance while 4,844 did not have such mutations (later in the text referred to as “wild type”).

Laboratories performing HIV-1 genotyping assays used identical analytical protocols and DNA sequencing equipment. From October 2001 to May 2004 and from September 2006 to December 2011, the program issued virtual phenotyping drug resistance reports (*Virtual*Phenotype or vircoTYPE *HIV-1*), as per a proprietary analytical protocol provided by Virco BVBA. A fragment of 1.8 kb encompassing the entire protease gene and the first 400 amino acids of the reverse transcriptase was amplified by nested RT-PCR and both strands were sequenced using a series of sequencing oligonucleotide primers and the BigDye Terminator Cycle Sequencing V 2.0 Ready Reaction kit (Life Technologies, Carlsbad, CA). Labeled DNA fragments were separated and detected using the ABI Genetic Analyzer 3100 or its upgraded version ABI 3130XL (Life Technologies, Carlsbad, CA). Sequence assembly and annotation were performed using the accompanying software SeqScape v2.5. Assembly parameters and rules for base calling were identical in the three laboratories. From June 2004 to August 2006, drug resistance was assessed using the TRUGENE HIV-1 assay from Bayer (Diagnostics Division, Bayer Corp., Tarrytown, N.Y.). DNA sequences and genotyping interpretation data from reports were directly incorporated into a centralized database at LSPQ.

### Antiretroviral prescriptions

The number of medical prescriptions of antiretroviral drugs per year in the Province of Quebec was graciously provided by IMS-Brogan.

### HIV-1 drug resistance data mining and compilations of results

As no nominal information is associated with test results, and because individuals seeking follow-up may use more than one health service provider, data extracted from the Quebec HIV-1 genotyping program database were refined as follows: only the earliest sequence per year among nucleotide sequences with more than 98% identity from individuals with the same date of birth and the same gender was included in analysis. This sequence identity threshold is routinely used to track cross-contamination, as suggested by Shafer *et al* [7]. According to this scheme, HIV genotyping assays were performed for 8,091 individuals from 2001 to 2011, of whom 5,834 had a single test performed during this period.

The Virco and Bayer Healthcare drug resistance reports use different interpretation rules. To alleviate the problem of comparing drug mutation patterns obtained using different algorithms and versions, all DNA sequences determined between 2001 and 2011 were reanalyzed in batch using the Sierra interface, the Stanford HIV Web Service (Version 1.0) and the Stanford HIVdb Program/Genotypic Resistance Interpretation Algorithm version 6.2.0. Resistance mutations were identified using the Stanford HIV drug resistance database (http://hivdb.stanford.edu/pages/download/resistanceMutations_handout.pdf, May 25, 2012). The frequency of resistance mutations per year was calculated as the ratio between the number of sequences containing resistance mutations on the number of tests performed.

### Statistical analyses

The presence of time trends in frequency of drug resistance mutations and in categories of viral load were assessed using univariate logistic regression. P<0.05 (in the odds ratio) indicates the presence of a statistically significant linear trend at the 95% level.

Multiple logistic regression controlling for lamivudine was used to estimate the association between the M184I/V mutation and emtricitabine over the time interval 2005–2011. Based on observed transition points in the trends of M184I/V, emtricitabine and lamivudine, a time lag of 2 years was assumed between the numbers of prescriptions and their effect on M184I/V frequency. Thus, the frequency of M184I/V from the years 2005–2011 was hypothesized to be associated with the number of prescriptions of emtricitabine from 2003–2009. All analyses were conducted in R 2.15.1 (R Development Core Team, 2012), [8].

## Results

### Viral load testing

**Figure 1:**
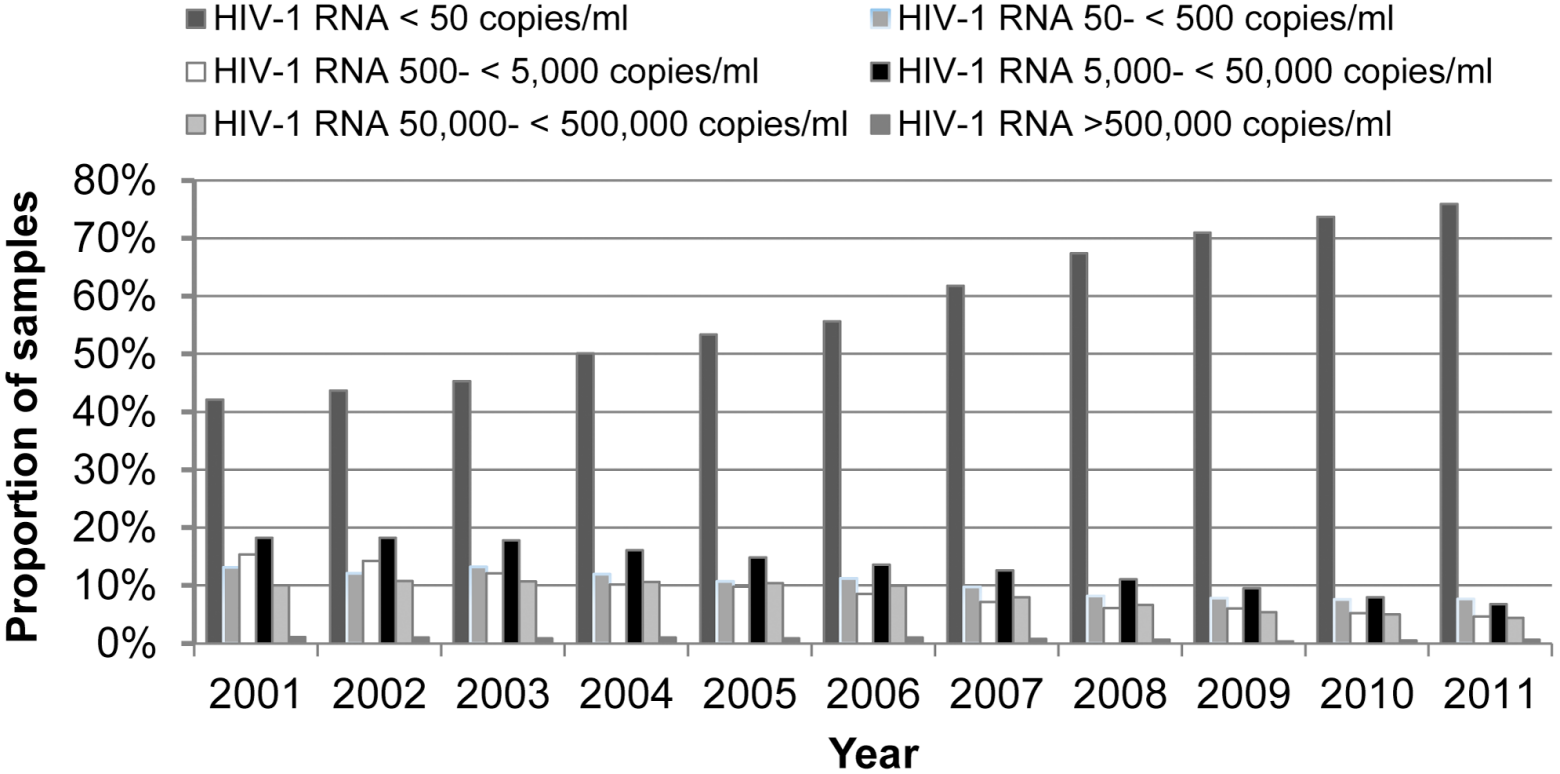
Time trends in the monitored viral load. Trends in the proportion of samples with suppressed [<1.7 log_10_ copies/ml (<50 copies/ml)] and unsuppressed plasma HIV viral loads, from 2001 to 2011. [*P* for trend <0.001 for all categories; OR corresponding to the change in frequency per year for: HIV RNA<50 copies/ml (OR = 1.17), HIV RNA 50–<500 copies/ml (OR = 0.93), HIV RNA 500–<5,000 copies/ml (OR = 0.87), HIV RNA 5,000–<50,000 copies/ml (OR = 0.90) and HIV RNA>500,000 copies/ml (OR = 0.92)].

The number of VL tests performed yearly in the province increased from 21,600 in 2001 to over 28,400 in 2011 (data not shown). During this period, the proportion of results reported as below the limit of detection (50 RNA copies/ml of plasma) showed a significant (P<0.001) increase, from 42.1% in 2001 to 75.9% in 2011 (**Figure 1**). Alternately, each category of ‘detectable VL’, between 500 and 5,000, 5,000 and 50,000, 50,000 and 500,000 and >500,000 RNA copies/ml of plasma declined significantly over the years (P<0.001).

### Trends in resistance mutation patterns

In 2001, 70% of sequences harbored resistance mutations and 30% exhibited wild type genotypes. This pattern had completely reversed in 2011 when 73% of amino acid sequences analyzed were from wild type viruses and 27% displayed at least one primary drug resistance mutation.

**Figure 2:**
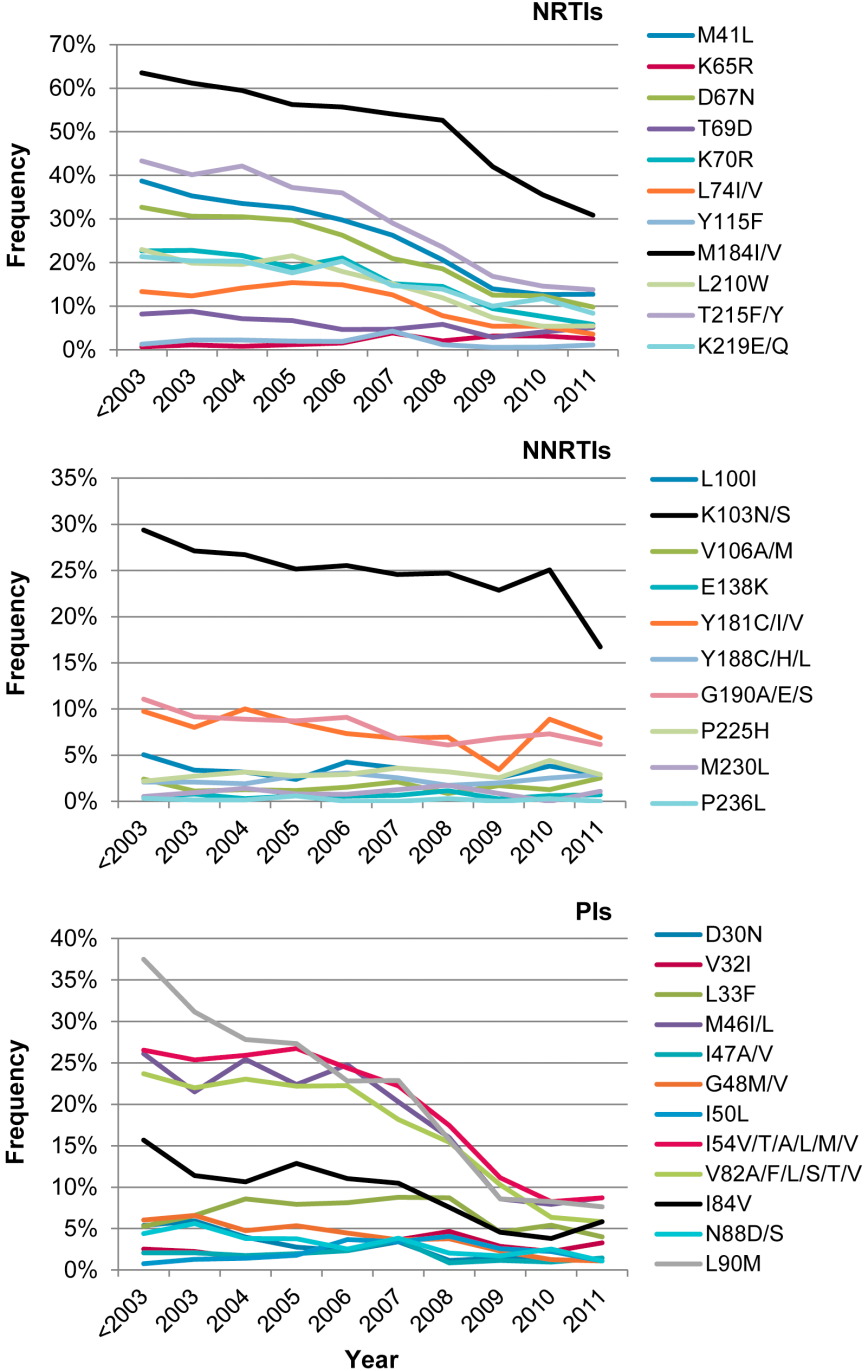
Time trends in frequency of antiretroviral resistance mutations among treatment-experienced patients. Frequency of antiretroviral resistance mutations at selected codons associated with decreased susceptibility to NRTIs, NNRTIs and PIs.

**Figure 3:**
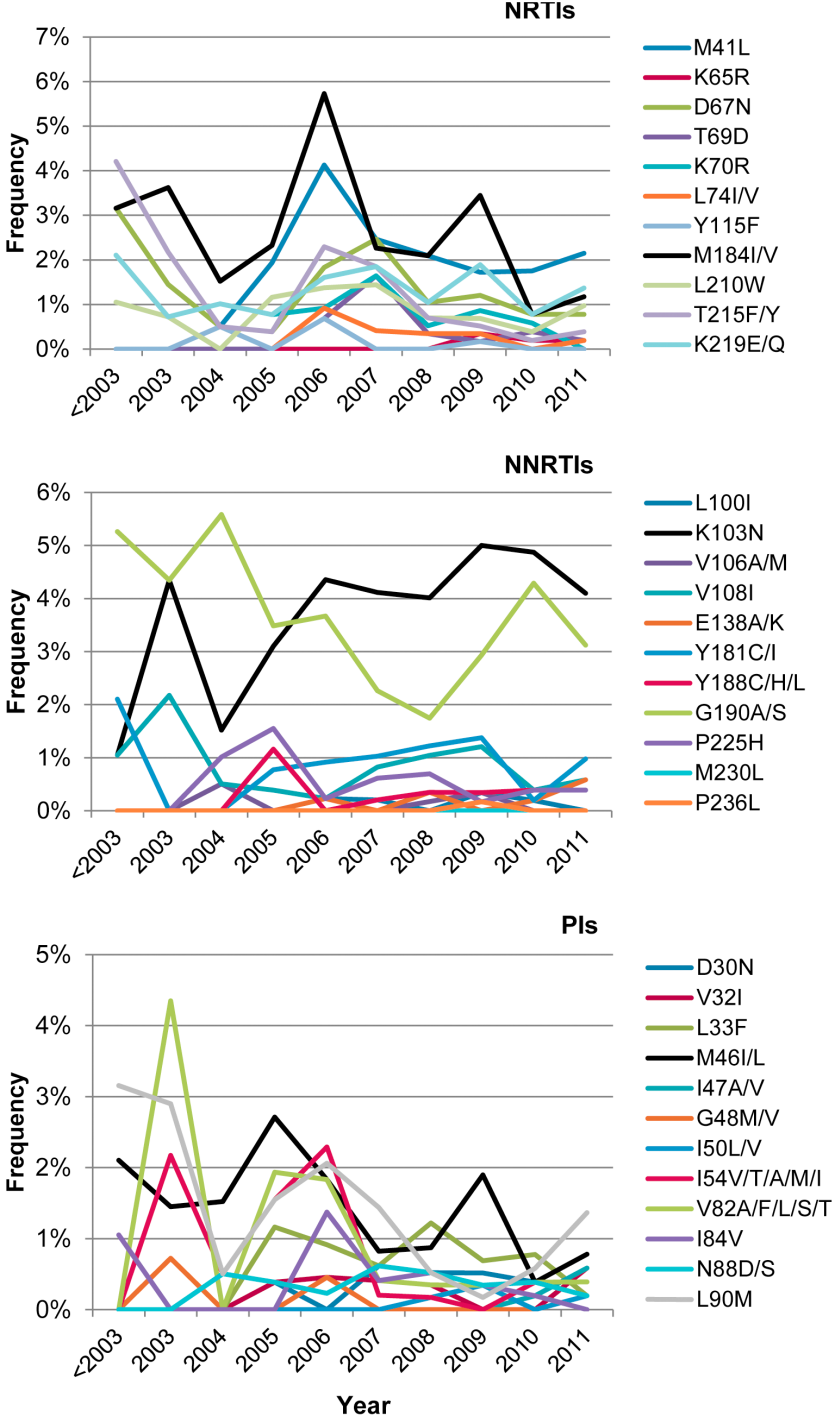
Time trends in frequency of antiretroviral resistance mutations among treatment-naïve patients. Frequency of antiretroviral resistance mutations at selected codons associated with decreased susceptibility to NRTIs, NNRTIs and PIs.

The frequency of PI, NRTI and NNRTI resistance mutations among treatment-experienced individuals continuously decreased from 2001 to 2011 (**Table 1**). The reduction in the frequency of any resistance mutation conferring resistance to PI (54.1% to 15.6%), NRTI (77.8% to 37.5%) and NNRTI (43.9% to 31.3%) was found to be statistically significant (P<0.001 for all cases). The reduction in the frequency of mutations associated with resistance to the three classes of ARV drugs (28.4% to 6.9%) was also statistically significant (P<0.001). In TN individuals (newly diagnosed and chronically infected), the frequency of transmitted drug resistance remained less than 13.1% over the period 2001–2011 (**Table 2**). No significant trends were observed in sequences carrying only NRTI or NNRTI-associated mutations while a slight decrease in the frequency of PI-associated mutations was observed over time (P<0.05). The frequency of mutations associated with resistance to the three classes of ARV drugs remained low among TN individuals, with an average frequency of 0.9%. The most common primary mutations associated with ARV resistance in TE individuals (**Figure 2**) were NRTIs: M184I/V (51.1%) and thymidine analog mutations (TAM) from TAM_1_ pathway (41L/210W/215Y; 17%), representing 76.2% of all TAM pathways (data not shown); NNRTIs: K103N (24.9%); PIs: L90 M (21.1%), V82 (16.8%), M46I/L (18.2%). No significant trend in frequency of ARV drug mutations was detected in TN individuals (**Figure 3**) although NNRTI-associated resistance mutations at amino acid positions 103 and 190 were most frequent at 3.65% and 3.67%, respectively. Since 2006, most of PI-associated resistance mutations were present in less than 2% of sequences determined for TN individuals.

**Table 1:** Treatment-experienced patients with drug resistance: frequency (%) of drug resistance over time, by drug class and their dual or triple combinations.

Year												
		<2003	2003	2004	2005	2006	2007	2008	2009	2010	2011	P-value
*Any	N	911	623	633	505	517	468	344	349	315	275	
**PI**	n	(54.1)	295	285	224	213	175	100	71	55	43	<0,001
	Frequency	493	(47.4)	(45.0)	(44.4)	(41.2)	(37.4)	(29.1)	(20.3)	(17.5)	(15.6)	
	95%-CI	50.8–57.3	43.5–51.2	41.2–49.0	40.0–48.7	36.9–45.5	33.1–41.9	24.4–34.0	16.3–24.6	13.3–21.6	11.6–20.0	
**NRTI**	n	709	456	454	344	333	292	199	167	131	103	<0,001
	Frequency	(77.8)	(73.2)	(71.7)	(68.1)	(64.4)	(62.4)	(57.8)	(47.9)	(41.6)	(37.5)	
	95%-CI	75.1–80.5	69.7–76.6	68.2–75.2	64.0–72.1	60.3–68.5	57.9–66.7	52.6–63.1	42.7–53.0	36.2–47.0	31.6–43.3	
**NNRTI**	n	400	251	261	191	197	167	129	110	121	86	<0,001
	Frequency	(43.9)	(40.3)	(41.2)	(37.8)	(38.1)	(35.7)	(37.5)	(31.5)	(38.4)	(31.3)	
	95%-CI	40.7–47.1	36.4–44.1	37.4–45.0	33.7–42.0	33.8–42.4	31.4–40.0	32.6–42.7	26.6–36.4	33.0–43.8	25.8–36.7	
*Mono*												
**PI**	N	4	2	3	7	2	3	1	2	1	5	NS
	Frequency	(0.4)	(0.3)	(0.5)	(0.4)	(0.6)	(0.3)	(0.6)	(0.6)	(0.3)	(1.8)	
	95%-CI	0.1–0.9	0–0.8	0–1.1	0.4–2.6	0–1	0–1.5	0–0.9	0–1.4	0–1.0	0.4–3.6	
**NRTI**	n	111	78	84	66	51	56	51	45	220	25	NS
	Frequency	(12.2)	(12.5)	(13.3)	(13.1)	(9.9)	(12.0)	(14.8)	(12.9)	(7.0)	(9.1)	
	95%-CI	10.1–14.4	10.0–15.2	10.7–16.0	10.3–16.0	7.4–12.6	9.2–15.0	11.0–18.6	9.5–16.6	4.4–9.8	5.8–12.7	
**NNRTI**	n	26	14	24	11	16	13	18	22	23	25	<0,001
	Frequency	(2.9)	(2.2)	(3.8)	(2.2)	(3.1)	(2.8)	(5.2)	(6.3)	(7.3)	(9.1)	
	95%-CI	1.9–4.0	1.1–3.5	2.4–5.4	1.0–3.6	1.7–4.6	1.5–4.3	2.9–7.6	4.0–8.9	4.4–10.2	5.8–12.7	
*Dual*												
**PI+NRTI**	n	227	144	139	100	103	87	40	37	15	18	<0,001
	Frequency	(24.9)	(23.1)	(22.0)	(19.8)	(19.9)	(18.6)	(11.6)	(10.6)	(4.8)	(6.5)	
	95%-CI	22.2–27.8	19.9–26.5	18.8–25.3	16.4–23.4	16.4–23.4	15.2–22.2	8.4–15.1	7.4–14.0	2.5–7.3	3.6–9.5	
**PI+NNRTI**	n	3	3	6	2	2	5	3	3	4	1	NS
	Frequency	(0.3)	(0.5)	(0.9)	(0.4)	(0.4)	(1.1)	(0.9)	(0.9)	(1.3)	(0.4)	
	95%-CI	0–0.8	0–1.1	0.3–1.7	0–1.0	0–1.0	0.2–2.1	0–2.0	0–2.0	0.3–2.5	0–1.1	
**NRTI+**	n	112	88	94	63	73	69	52	56	59	41	0,01
**NNRTI**	Frequency	(12.3)	(14.1)	(14.8)	(12.5)	(14.1)	(14.7)	(15.1)	(16.0)	(18.7)	(14.9)	
	95%-CI	10.2–14.5	11.4–16.9	12.2–17.7	9.7–15.4	11.2–17.2	11.5–17.9	11.3–18.9	12.3–20.1	14.6–23.2	10.9–19.3	
*Triple*												
**PI+NRTI+**	n	259	146	137	115	106	80	56	29	35	19	<0,001
**NNRTI**	Frequency	(28.4)	(23.4)	(21.6)	(22.8)	(20.5)	(17.1)	(16.3)	(8.3)	(11.1)	(6.9)	
	95%-CI	25.6–31.4	20.2–26.8	18.5–25.0	19.2–26.5	17.0–24.0	13.7–20.5	12.5–20.3	5.4–11.2	7.6–14.6	4.0–10.2	

n: number of resistance tests (first test/patient per year) presenting with mutations associated to resistance; N: Total number of patients with resistance test in that period; CI: confidence interval; Significant effects (P≤0.05) are bolded. NS: not significant.

**Table 2:** Treatment-naïve patients with drug resistance: frequency (%) of drug resistance over time, by drug class and their dual or triple combinations.

Year												
		<2003	2003	2004	2005	2006	2007	2008	2009	2010	2011	P-value
	N	95	138	197	258	438	486	571	578	513	511	
**Overall drug**	n	12	24	24	33	61	56	66	88	65	56	NS
**resistance**	Frequency	(12.6)	(17.4)	(12.2)	(12.8)	(13.9)	(11.5)	(11.6)	(15.2)	(12.7)	(11)	
	95%-CI	6.3–20	11.6–23.9	7.6–16.8	8.9–17.1	10.7–17.4	8.8–14.4	8.9–14.2	12.3–18.2	9.9–15.6	8.4–13.7	
*Any												
**PI**	n	4	8	6	13	18	16	15	15	9	13	0.003
	Frequency	(4.2)	(5.8)	(3.0)	(5.0)	(4.1)	(3.3)	(2.6)	(2.6)	(1.8)	(2.5)	
	95%-CI	1.1–8.4	2.2–10.1	1.0–5.6	2.7–7.8	2.3–6.2	1.9–4.9	1.4–4.0	1.4–4.0	0.8–2.9	1.4–3.9	
**NRTI**	n	6	7	5	12	38	26	25	36	17	18	NS
	Frequency	(6.3)	(5.1)	(2.5)	(4.7)	(8.7)	(5.3)	(4.4)	(6.2)	(3.3)	(3.5)	
	95%-CI	2.1–11.6	1.4–8.7	0.5–5.1	2.3–7.4	6.2–11.4	3.5–7.4	2.8–6.1	4.3–8.3	1.9–4.9	2.0–5.3	
**NNRTI**	n	8	16	15	20	40	33	42	58	48	46	NS
	Frequency	(8.4)	(11.6)	(7.6)	(7.8)	(9.1)	(6.8)	(7.4)	(10.0)	(9.4)	(9.0)	
	95%-CI	3.2–14.7	6.5–17.4	4.1–11.7	4.7–11.2	6.6–11.9	4.7–9.1	5.3–9.6	7.6–12.6	6.8–11.9	6.7–11.5	
*Mono*												
**PI**	n	2	4	5	5	2	5	8	9	3	5	0.048
	Frequency	(2.1)	(2.9)	(2.5)	(1.9)	(0.5)	(1.0)	(1.4)	(1.6)	(0.6)	(1.0)	
	95%-CI	0–5.3	0.7–5.8	0.5–5.1	0.4–3.9	0–1.1	0.2–2.1	0.5–2.5	0.7–2.6	0–1.4	0.2–2.0	
**NRTI**	n	1	3	3	4	15	14	12	18	10	3	NS
	Frequency	(1.1)	(2.2)	(1.5)	(1.6)	(3.4)	(2.9)	(2.1)	(3.1)	(1.9)	(0.6)	
	95%-CI	0–3.2	0–5.1	0–3.6	0.4–3.1	1.8–5.3	1.4–4.5	1.1–3.3	1.7–4.7	0.8–3.3	0–1.4	
**NNRTI**	n	4	11	14	16	19	23	33	42	44	32	NS
	Frequency	(4.2)	(8.0)	(7.1)	(6.2)	(4.3)	(4.7)	(5.8)	(7.3)	(8.6)	(6.3)	
	95%-CI	1.1–8.4	3.6–13.0	3.6–10.7	3.5–9.3	2.5–6.4	2.9–6.8	4.0–7.7	5.2–9.5	6.2–11.1	4.3–8.4	
*Dual*												
**PI+NRTI**	n	1	1	1	4	4	4	4	3	4	2	NS
	Frequency	(1.1)	(0.7)	(0.5)	(1.6)	(0.9)	(0.8)	(0.7)	(0.5)	(0.8)	(0.4)	
	95%-CI	0–3.2	0–2.2	0–1.5	0.4–3.1	0.2–1.8	0.2–1.6	0.2–1.4	0–1.2	0.2–1.6	0–1.0	
**PI+NNRTI**	n	0	2	0	0	2	2	0	1	1	1	NS
	Frequency	(0)	(1.4)	(0)	(0)	(0.5)	(0.4)	(0)	(0.2)	(0.2)	(0.2)	
	95%-CI	0–0	0–3.6	0–0	0–0	0–1.1	0–1.0	0–0	0–0.5	0–0.6	0–0.6	
**NRTI+NNRTI**	n	3	2	1	0	9	3	6	13	2	8	NS
	Frequency	(3.2)	(1.4)	(0.5)	(0)	(2.1)	(0.6)	(1.1)	(2.2)	(0.4)	(1.6)	
	95%-CI	0–7.4	0–3.6	0–1.5	0–0	0.9–3.4	0–1.4	0.4–1.9	1.2–3.5	0–1.0	0.6–2.7	
*Triple*												
**PI+NRTI+NNRTI**	n	1	1	0	4	10	5	3	2	1	5	NS
	Frequency	(1.1)	(0.7)	(0)	(1.6)	(2.3)	(1.0)	(0.5)	(0.3)	(0.2)	(1)	
	95%-CI	0–3.2	0–2.2	0–0	0.4–3.1	0.9–3.9	0.2–2.1	0–1.2	0–0.9	0–0.6	0.2–2.0	

n: number of resistance tests (first test/patient per year) presenting with mutations associated to resistance; N: Total number of patients with resistance test in that period; CI: confidence interval; Significant effects (P≤0.05) are bolded. NS: not significant.

The proportion of sequences belonging to subtype B represented 92.4% in 2001 and gradually declined to 85.0% in 2011 (data not shown).

### Impact of current treatments on frequency of specific mutations

**Figure 4:**
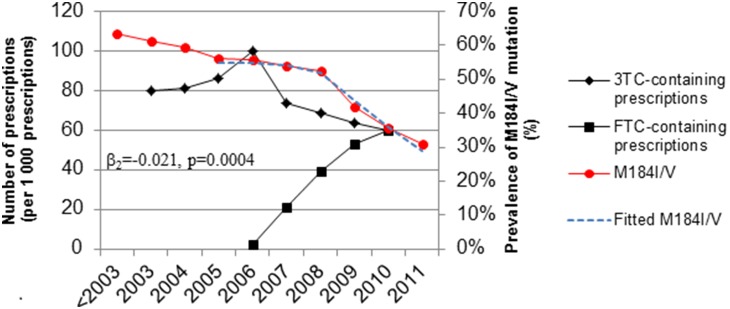
Temporal trends of lamivudine (3TC) or emtricitabine (FTC)-containing prescriptions (black) with the frequency of the M184I/V mutation (red). Association between M184I/V mutation and emtricitabine over the time interval 2005–2011, controlling for lamivudine, was estimated using the multivariate logistic regression model: logit(PM184)∼β0 + β1 × 3TC + β2 × FTC. The slope parameter β2 represents the log odds ratio for the change in M184I/V frequency associated with a one unit (i.e. 1 per 1000) increase in emtricitabine prescriptions. The p-value<0.05 indicates a statistically significant linear association at the 95% level.

We observed a decrease in the frequency of the drug resistance mutations M184I/V over time (**Figure 4**). M184I/V mutations abruptly decreased in the year 2009. This transition point is hypothesized to be associated with replacement of lamivudine - by emtricitabine -based prescriptions that became substantial two years earlier, in 2007. Multiple logistic regression analysis indicated that the introduction of co-formulated emtricitabine/tenofovir or emtricitabine/tenofovir/efavirenz was positively associated with the decrease of M184I/V mutations (p≈0.0004). The logistic regression model, logit(M184I/V)∼β0 + β1× lamivudine + β2 × emtricitabine, controlled for the presence of lamivudine, implemented a lag time of two years between mutation and prescription prevalence, and accounted for overdispersion. The slope parameter β2 was estimated to be ≈−0.021 (p≈0.0004) and reflects the strength of association between M184I/V and emtricitabine co-formulations.

## Discussion

This study is the first to describe trends in HIV drug resistance mutation frequency and HIV-1 VL, among treatment-naïve (TN) and treatment experienced (TE) HIV-infected patients over 11 years (2001–2011) of a HIV surveillance program. The past decade has been marked by the availability of more efficient treatments for HIV/AIDS leading to a spectacular decline in mortality and morbidity [9], increased life expectancy [10], and a net diminution of HIV transmission [1], [11], [12]. This has been accompanied by decreased frequency of HIV drug resistance [13], [14].

Between 2001 and 2011, the proportion of HIV-1 positive individuals in the province of Québec exhibiting undetectable VL increased substantially (from 42.1% in 2001 to 75.9% in 2011). Viral suppression not only prevents HIV transmission [11], [15], [16] but also transmission of drug resistance [17]. Before 2003, therapeutic failure was the main clinical justification for requesting a genotyping test (78%), whereas in 2011, ART initiation (baseline test for chronically infected individuals initiating ART) became the major reason for such requests. In TN individuals (newly diagnosed and chronically infected), the overall frequency of transmitted drug resistance (TDR) to any ARV class was 13.1% ranging between 11% and 17.4%. Overall, TDR by ARV class was 8.7% (NNRTIs), 5% (NRTIs), and 3.5% (PIs). Similar rates of transmitted drug resistance mutations, varying from 5.6 to 25%, have been described in other Northern countries in acutely or recently infected persons [18]–[26]. In TN individuals, M41L (2.1%), M184I/V (2.6%), K103N/S (3.65%) and G190A/S (3.67%) were the most common mutations detected.

We previously described a transmission chain of G190A-containing infections among primary/recently HIV-infected individuals, between 2001 and 2007 [14]. Our results show that since 2004, the frequency of K103N/S increased from 1.5% before 2003 to 4.1% in 2011 while G190A/S frequency decreased from 5.6% to 3.1%. This change in NNRTI mutation trends suggests that the profile of Quebec’s primary/recent HIV infected population may have evolved towards favoring K103N variants as recently shown by Ruelle *et al.* [27], who studied a subtype B HIV variant harboring K103N related to a cluster of 29 virus sequences.

In TE individuals, the frequency of drug resistance mutations associated with the three major classes of ARV was in constant decline from 2001 to 2011. The frequency of the most common NRTI-associated resistance mutations, M184 V/I, decreased by 20% in 2011 as compared to the 2001–2003 period. This decrease has been maintained as new co-formulated regimens were offered to patients (emtricitabine/tenofovir; 2006) and (emtricitabine/tenofovir/efavirenz; 2007) and is doubtless due in large part to increased adherence to therapy that is associated with these co-formulations and to the long half-lives of the drugs involved. Recent studies have described a lower frequency of M184 V mutations in patients with VL>100,000 copies/ml treated with emtricitabine/tenofovir compared to patients treated with lamivudine/tenofovir [28]–[30]. This decline has continued as fixed-dosed ARVs (emtricitabine/tenofovir/efavirenz; 2007) as well as new molecules such as etravirine (2008), that do not show cross-resistance with the previous generation of drugs, have become available.

Our study shows that both transmitted and acquired drug resistance mutations have been in decline along with HIV VL, suggesting the effectiveness of current antiretroviral treatments. Although we demonstrate that levels of transmitted drug resistance mutations were relatively low over time, the persistence of a transmitted resistance in a subset of patients justifies that screening should continue for primary drug resistance in all newly HIV-diagnosed individuals [31]. Mutations such as K103N, which do not impact significantly on viral fitness, can persist in the absence of drug pressure [32]–[34] and interfere with NNRTI-based treatment effectiveness [35], [36]. Numerous studies have demonstrated that primary HIV infection plays a central role in the spread of the HIV epidemic [32], [37], [38]. In Québec, we have demonstrated that 50 percent of all new cases of HIV infection can be attributed to recently infected people [11]. All of these observations argue in favour of a sustained and targeted monitoring of patients newly infected with HIV-1.
